# Multidrug-Resistant Methicillin-Resistant *Staphylococcus aureus* Associated with Hospitalized Newborn Infants

**DOI:** 10.3390/diagnostics13061050

**Published:** 2023-03-09

**Authors:** Ching Hoong Chew, Chew Chieng Yeo, Ainal Mardziah Che Hamzah, Esra’a I. Al-Trad, Sherry Usun Jones, Kek Heng Chua, Suat Moi Puah

**Affiliations:** 1 Faculty of Health Sciences, Universiti Sultan Zainal Abidin, Kuala Terengganu 21300, Malaysia; 2 Centre for Research in Infectious Diseases and Biotechnology (CeRIDB), Faculty of Medicine, Universiti Sultan Zainal Abidin, Kuala Terengganu 20400, Malaysia; 3 Department of Biomedical Science, Faculty of Medicine, Universiti Malaya, Kuala Lumpur 50603, Malaysia

**Keywords:** antibiotic susceptibility testing, biofilm, methicillin-resistant *Staphylococcus aureus*, multidrug resistance, neonates, whole genome sequencing

## Abstract

Multidrug resistance (MDR) is a significant challenge in healthcare management, and addressing it requires a comprehensive approach. In this study, we employed a combination of phenotypic and genotypic approaches, along with whole genome sequencing (WGS) to investigate five hospital-associated MDR methicillin-resistant *Staphylococcus aureus* (MRSA) strains that were isolated from newborn infants. Our analysis revealed the following for the MDR-MRSA strains: SauR31 was resistant to three antimicrobial classes; SauR12, SauR91 and SauR110 were resistant to four antimicrobial classes; and SauR23 exhibited resistance to seven classes. All the MDR-MRSA strains were capable of producing slime and biofilms, harbored SCC*mec* type IV, and belonged to different *spa* types (t022, t032, and t548), with varying profiles for microbial surface components recognizing adhesive matrix molecules (MSCRAMMs) and virulence genes. The WGS data for the MDR SauR23 and SauR91 strains revealed that most of the antimicrobial resistance genes were present in the chromosomes, including *blaZ*, *mecA*, *norA*, *lmrS*, and *sdrM*, with only the *ermC* gene found in a small (<3 kb) plasmid. The presence of MDR-MRSA strains among neonates raises public concern, hence implementation of multifaceted interventions is recommended to address this issue. In addition, metadata is needed to improve the investigation of antimicrobial resistance genes in MDR isolates.

## 1. Introduction

The first 28 days of life are the most vulnerable for preterm infants, who are at high risk of life-threatening infections and mortality [1]. Among the most significant nosocomial pathogens, methicillin-resistant *Staphylococcus aureus* (MRSA) is of particular concern for neonatal infections, with colonization rates ranging from 3.9% to 32% [2]. In the hospital environment, MRSA is likely to be acquired through the birth canal and/or through contact with people such as parents, healthcare workers, visitors, or contaminated hospital environments [2,3]. Based on a meta-analysis report, infants who are colonized with MRSA are approximately five times more likely to develop MRSA infections compared to those who are not colonized [4]. MRSA infections can manifest in various forms, including skin and soft tissue infections, respiratory tract infections, pneumonia, bacteremia, osteomyelitis, and septic arthritis [2]. MRSA produces multiple virulence factors that facilitate its adhesion, invasion, and persistence within the host. For example, Panton–Valentine leukocidin (PVL) is responsible for neutrophil lysis and is associated with soft tissue infections and necrotizing pneumonia [5]. Adhesion of the bacteria to the host’s extracellular matrix factors (e.g., laminin, elastin, fibrinogen, fibronectin, and collagen) is facilitated by microbial surface components recognizing adhesive matrix molecules (MSCRAMMs), which also participate in biofilm formation to protect the bacteria from the host’s immune system and antibiotic treatment [6]. The bacterial invasion is facilitated by exfoliative toxins while its persistence is associated with enterotoxins, and toxic shock syndrome toxin-1, which act as superantigens to suppress the host immune response [7]. Overall, the staphylococcal infection process involves a combination of numerous virulence factors.

According to the 2019 Center for Disease Control and Prevention (CDC) report, antimicrobial resistance (AMR) is one of the most important current threats to public health today [8]. The recent analysis of the global impact of AMR reported that AMR was directly responsible for an estimated 1.27 million deaths worldwide and associated with 4.95 million deaths [9]. Another serious issue related to AMR is increasing multidrug resistance (MDR) in which pathogens develop resistance to multiple antimicrobial agents simultaneously. Several recent investigations have reported the emergence of MDR bacterial pathogens from different origins that increase the necessity of antibiotic stewardship, or the proper use of antibiotics in the healthcare system [10,11,12,13]. Among 20 pathogens categorized under four sections of antibiotic resistance threats in the 2019 CDC report, MRSA was listed under the section of serious threats [8]. Due to multidrug resistance, therapeutic options for patients with MRSA infections are limited and are a challenge to physicians. Thus far, no MRSA isolate in Malaysia has been reported to be fully resistant to the drug of last resort, vancomycin [14,15]. According to the third edition of the Malaysian National Antibiotic Guideline 2019, the drugs to be used for treating MRSA infections in Malaysia are clindamycin, cloxacillin, daptomycin, doxycycline, gentamicin, linezolid, rifampin, trimethoprim/sulfamethoxazole and vancomycin based on different clinical presentation conditions [16]. Macrolide–lincosamide–streptogramin B (MLS_B_) is another significant AMR phenotype for staphylococci which, when acquiring or developing resistance to MLS_B_ antibiotics, can be manifested either as constitutive (cMLS_B_) or inducible (iMLS_B_). iMLS_B_ is also known as inducible clindamycin resistance, since using conventional disk diffusion tests, the isolate appears as clindamycin susceptible, but in the presence of the macrolide erythromycin, the isolate is induced leading to clindamycin resistance, therefore increasing the risk of clinical treatment failure. iMLS_B_ can only be detected using the D-test, in which a D-shaped distorted inhibition zone is observed around clindamycin under the in vitro effect of erythromycin. Identification of iMLS_B_ is thus crucial for the proper management of *S. aureus* infections [17].

Neonatal MRSA infections not only have high mortality and morbidity rates but also cause long-term adverse effects on neonates. Based on the latest Malaysian National Antibiotic Resistance Surveillance Report (NSAR), MRSA rates were approximately 18% in 2016 and 19.8% in 2017, with a slight decrease to 16.1% in 2018, 14.9% in 2020, and a significant drop to 7% in 2021 [18]. The earliest reported cases of MRSA infections in Malaysian neonates date back to 1984, when 6% (53/858) of infants in the special care nursery of Kuala Lumpur Maternity Hospital were colonized with MRSA [19]. Unfortunately, epidemiological data on MRSA in Malaysian neonates are scarce and limited to one or two centers of study. For instance, in the 1990s, Cheong et al. (1994) found a high number of MRSA isolates (26.3%, 142/539) in the pediatric and special care nurseries of two Kuala Lumpur hospitals between August 1990 and November 1991 [20]. In the late 2000s, Kuala Lumpur Hospital reported an MRSA rate of 9.2% (174/1887) in pediatrics patients between October 2007 and September 2008 [21]. A more recent study conducted in Kuala Lumpur Hospital from January 2016 to December 2017 showed that 33% of the 3030 isolates from pediatric wards were MRSA [22]. Additionally, NSARs reported that over 200 MRSA isolates were collected annually from neonatal intensive care units and/or pediatric intensive care units in 2017–2019, with a decrease to below 100 isolates from 2020–2021 [23]. Collectively, these data indicate that MRSA remains a significant problem for hospitalized neonates in Malaysia, especially amongst preterm and newborn neonates. Urgent and appropriate medical management is required to prevent and control MRSA infections in this vulnerable population.

The lack of data from Malaysian hospitals regarding MRSA infections, particularly among neonates, leads us to the main objective of the present study, which is to characterize five hospital-associated MDR-MRSA isolated strains from infants collected from Hospital Sultanah Nur Zahirah (HSNZ), the main tertiary hospital in Terengganu, Malaysia from July 2016 to June 2017. Phenotypic characterization was performed for antibiotic susceptibility against 18 antimicrobial classes including determining the MLSB phenotype, and investigating the capability of biofilm and slime formation for each isolate. Genotypic characterization included the screening of three staphylococcal molecular types via *spa* typing, *agr* typing and staphylococcal chromosomal cassette *mec* (SCC*mec*) typing, and determining the carriage of 19 virulence genes and 15 MSCRAMMs genes using polymerase chain reaction (PCR) assay. We then selected two MDR isolates, SauR23 (resistance to seven antimicrobial classes) and SauR91 (resistance to four antimicrobial classes) for further characterization using whole genome sequencing (WGS).

## 2. Materials and Methods

### 2.1. Staphylococcus aureus Isolates Collection, Identification and Clinical Background

Five hospital-associated MRSA isolates from hospitalized neonates were identified via routine procedures at the Microbiology Laboratory in HSNZ, Kuala Terengganu (July 2016 to June 2017). HSNZ is a tertiary referral hospital with a total of 45 wards and 1127 beds, including Special Care Neonate and Neonatal Intensive Care wards. MRSA were isolated using commercial blood agar (Thermo Scientific, Winsford, United Kingdom). Presumptive MRSA colonies were selected for Gram stain (Thermo Scientific, Winsford, UK) examination under compound light microscope (Nikon, Tokyo, Japan) and Staphaurex™ latex agglutination test (Thermo Scientific, Winsford, UK). Clinical isolates and patient data were collected with the approval of Medical Research and Ethics Committee (NMRR-MREC), Ministry of Health Malaysia, under the National Medical Research Registry protocol number: NMRR-15-2369-28130 (IIR).

In the research laboratory, a 15% glycerol stock culture was prepared upon the collection of isolates from HSNZ. The *S. aureus* was subcultured onto mannitol salt agar (Thermo Scientific, Winsford, UK) using the standard four-way streaking method to obtain a single pure colony. The single pure colony was then subcultured in LB broth (Thermo Scientific, Winsford, UK) and incubated overnight with shaking. The LB broth culture was centrifuged at 5000× *g* for 10 min and the supernatant was replaced with fresh prepared LB broth containing 15% (*v/v*) glycerol which was then stored at −80 °C until further manipulation. Molecular PCR targeting the *nuc* and *mecA* genes was carried out for MRSA confirmation as described in [17].

General clinical data for the isolates were collected from the Data Unit of HSNZ. The demographic of the five patients is shown in Table 1. Four of the validated MRSA isolates (which were designated SauR12, SauR23, SauR31, and SauR110) were obtained from eye and pus specimens of the infected neonates and their treatment outcomes were positive. Another MRSA (SauR91) was isolated from the respiratory specimen of a neonate with congenital abnormality hospitalized with lung abscess, chronic lung disease, airway malacia, and diabetic embryopathy. The patient succumbed to the infection at six months of age and after 183 days of hospitalization since birth.

### 2.2. Antibiotic Susceptibility Testing

The isolates were subjected to antibiotic susceptibility testing (AST) towards 18 antimicrobial classes encompassing 26 antimicrobial agents, i.e., β-lactams (penicillin, oxacillin, cefoxitin, cefoperazone); fluoroquinolones (ciprofloxacin, moxifloxacin); macrolides (erythromycin); lincosamides (clindamycin); aminoglycosides (gentamicin, amikacin); folate inhibitor (co-trimoxazole); fusidanes (fusidic acid); tetracyclines (tetracycline, doxycycline, minocycline); glycylcyclines (tigecycline); phenicols (chloramphenicol); monoxycarbolic acid (mupirocin); ansamycin (rifampin); aminocoumarins (novobiocin); glycopeptides (vancomycin, teicoplanin); oxazolidinones (linezolid); phosphonic acid (fosfomycin); streptogramin B (quinupristin-dalfopristin); and anti-MRSA cephalosporins (ceftaroline) (Thermo Scientific™ Oxoid™, Winsford, UK) using the Kirby–Bauer disc diffusion method as previously described [17]. The susceptibility categorization was based on the Clinical and Laboratory Standards Institute (CLSI) and the European Committee on Antimicrobial Susceptibility Testing (EUCAST) guidelines [24,25]. *S. aureus* is defined as multidrug resistant (MDR) when the isolate is resistant to at least one agent in three or more antibiotic categories while extensively drug-resistant (XDR) isolate is indicated by resistance to at least one agent in all but two or fewer antibiotic categories. A pandrug-resistant (PDR) bacterium, on the other hand, displays resistance to all agents in all antimicrobial categories [26]. The D-test was also performed to determine the macrolide–lincosamide–streptogramin B (MLSB) resistance phenotype with the placement of clindamycin (2 μg) and erythromycin (15 μg) antibiotic discs next to each other at a distance of 15–26 mm apart [17].

### 2.3. Spa Typing, Agr Typing, SCCmec Typing, and Detection of Adhesin (MACRAMMs) and Virulence Genes

DNA amplification of *spa* locus, *agr* groups, 15 MSCRAMM-associated genes (*bap*, *bbp*, *clfA*, *clfB*, *cna*, *eno*, *ebps*, *fib*, *fnbA*, *fnbB*, *icaAD*, *icaBC*, *pls*, *sasC*, and *sasG*) and 19 virulence genes including staphylococcal enterotoxin genes (*sea*, *seb*, *sec*, *seg*, *seh*, *sei*, *sel*, *sem*, *sen*, *seo*, and *ser*), toxic shock syndrome toxin-1 gene (*tst*), exfoliative toxin gene (*eta*), leukocidin genes (*lukED* and *lukPV*), and hemolysin genes (*hla*, *hlb*, *hld*, and *hlg*) was performed using primers and conditions as described in previous published studies [27,28,29]. The isolates were also screened for their SCC*mec* type by PCR amplification with standard protocols [30].

### 2.4. Congo Red Assay and Biofilm Formation Assay

Detection of slime production was performed using brain heart infusion-based (Becton Dickinson, Sparks, NV, USA) Congo red agar (MP Biomedicals, Steven Hills, NSW, Australia) (CRA) supplemented with 5% sucrose (MP Biomedicals, NSW, Australia). The slime-producing *Staphylococcus epidermidis* ATCC 35,984 and non-slime producing *Staphylococcus hominis* ATCC 35,982 were used as positive and negative controls, respectively. The CRA plate was incubated at 37 °C for 24 h and 48 h. Results were classified based on colony color as follows: black (strong), a mixture of black and red (moderate), and red (weak) [31].

Detection of biofilm formation was performed using a microtiter plate with crystal-violet staining. The biofilm-producing *Staphylococcus aureus* ATCC 35,556 and non-biofilm-producing *Staphylococcus epidermidis* ATCC 12,228 were used as positive and negative controls, respectively. Bacterial cultures were seeded on a microtiter plate and incubated at 37 °C for 24 h. Bacterial growth was measured spectrophotometrically at 600 nm (Implen, München, Germany) on the next day; the adhered cells were fixed with absolute ethanol, stained by crystal violet (MP Biomedicals, NSW, Australia), solubilized using acetic acid, and measured at 570 nm. After that, a specific biofilm formation (SBF) index was calculated [32].

### 2.5. Real-Time Impedance-Based Assay

Two MDR-MRSA isolates, SauR23 and SauR91, were selected for further characterization for real-time biofilm formation and WGS (Section 2.6) due to their unique properties: (i) SauR23 exhibited resistance to seven antimicrobial classes, harbored the highest number of virulence genes and demonstrated strong production for slime and biofilms; (ii) SauR91 caused a fatal case, exhibited resistance to four antimicrobial classes, and demonstrated moderate production for slime and biofilms. To monitor biofilm development in real time, a mid-log phase culture was inoculated into the E-plate (Agilent Technologies, CA, USA) while trypticase soy broth (Becton Dickinson, Sparks, NV USA) was used as the background impedance. The E-plate was incubated at 37 °C on the xCELLigene Real-Time Cell Analyzer (Agilent Technologies, CA, USA) and monitored every 10 min time intervals for 48 h [31]. Impedance measurement was displayed as cell index (CI) value, which corresponded to the measurement of total biofilm biomass of adherent bacteria to the bottom gold microelectrodes. Three independent experiments were performed in triplicates and the average CI ± standard deviation was calculated.

### 2.6. Whole Genome Sequencing and Bioinformatics Analysis

WGS and bioinformatics analysis were carried out as described in [33]. Genomic DNA was extracted using a Presto™ Mini gDNA Bacteria Kit (Geneaid Biotech Ltd., Taiwan) based on the manufacturer’s instructions with the addition of a lysostaphin (Sigma-Aldrich, Saint Louis, MO, USA) digestion step. The samples were then sent to a commercial service provider (Novogene Co., Ltd., Singapore) for WGS using an Illumina HiSeq-PE150 high-throughput sequencer platform with a paired-end sequencing strategy. Sequence data were de novo assembled using Unicycler assembler (v0.5.0) [34]. PATRIC RASTtk-enabled Genome Annotation Service [35] was used to annotate the assembled genome. A circular genome map was generated using CGView [36] at Proksee (https://proksee.ca/ (accessed on 1 December 2022)). The antimicrobial gene carriage was determined by ResFinder 4.1 [37,38] available at the website Center for Genomic Epidemiology (CGE) database (https://www.genomicepidemiology.org/services/ (accessed on 1 January 2023)) and also the CARD Resistance Gene Identifier [39] available at https://card.mcmaster.ca/analyze/rgi (accessed on 1 January 2023). PlasmidSPAdes version v3.13.0 [40] and PlasmidFinder software version 2.1 [41], also available at the CGE database, were used to identify potential plasmids from the assembled contigs. Molecular typing analysis such as spaTyper (for *spa* typing), multilocus sequence typing (MLST), SCCmecFinder, and VirulenceFinder are also available at the CGE.

## 3. Results

In this study, we present a comprehensive analysis of five MRSA isolates, which covers (i) phenotypic profiles, including antimicrobial resistance profiles, as well as their ability to form slime and biofilm-forming and (ii) genotypic profiles of *spa*, *agr*, *SCCmec*, MSCRAMMs, and virulence genes. Furthermore, we demonstrate the real-time formation of biofilm for two MDR isolates (SauR23 and SauR91) and their draft genome sequences.

### 3.1. Phenotypic and Genotypic Characteristics of the Recovered Isolates

In the university’s research laboratory, all five MRSA isolates were recovered from archival glycerol stock and showed the appearance of golden colonies in blood agar, and yellow colonies (mannitol fermenter) on mannitol salt agar. With Gram staining, the bacterial cells were Gram-positive cocci in clusters. The isolates were also coagulase positive as well as cefoxitin resistant, and PCR showed the presence of the *nuc* and *mecA* genes, which were indicative of MRSA [17].

### 3.2. Antibiotic Susceptibility Testing (AST)

AST results showed that all five MRSA isolates were resistant to 12 antibiotics while remaining susceptible to 14 antibiotics (namely, ceftaroline, chloramphenicol, co-trimoxazole, doxycycline, fosfomycin, linezolid, minocycline, mupirocin, novobiocin, quinupristin-dalfopristin, rifampin, teicoplainin, tigecycline, and vancomycin) (Table 2). All five MRSA isolates were resistant to β-lactams (penicillin, oxacillin, cefoxitin, cefoperazone), macrolides (erythromycin), and lincosamides (clindamycin). Thus, all MRSA isolates were multidrug resistant following the criteria of Magiorakos et al. [26] whereby SauR31 showed resistance to three antimicrobial classes, while SauR12, SauR91, and SauR110 showed resistance to four antimicrobial classes, and SauR23 exhibited the highest resistance of up to seven antimicrobial classes. All five isolates were D-test positive, indicative of the iMLS_B_ phenotype (or inducible clindamycin resistance).

### 3.3. Profiling of Spa, Agr, SCCmec, MLS_B_, Adhesin (MSCRAMMs), and Virulence Genes

**Figure 1:**
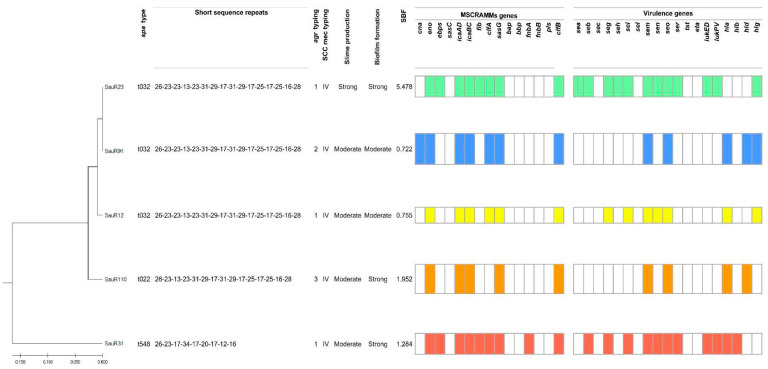
Dendrogram showing the *spa* types, *agr* types, SCC*mec* types, slime and biofilm production, profiles of MSCRAMMs, and virulence genes.

The UPGMA dendrogram based on the carriage of MSCRAMMs and virulence genes showed the isolates could be divided into two major clades, one clade containing SauR31 which belonged to *spa* type t548, and another clade consisting of two lineages, i.e., SauR12, SauR23, and SauR91, which belonged to *spa* type t032, while SauR110 belonged to *spa* type t022 (Figure 1). The *spa* type t022 had one deletion in the repeat succession compared with *spa* type t032 and shared a similarity of 98.5%. Three isolates belonged to *agr* group 1 (SauR12, SauR23, and SauR31), one isolate belonged to *agr* group 2 (SauR91), and another isolate belonged to *agr* group 3 (SauR110). SCC*mec* typing revealed that all five isolates carried SCC*mec* type IV. Of the 15 MSCRAMMs, *eno*, *icaAD*, *icaBC*, *sasG,* and c*lfB* were detected in all five isolates while SauR31 carried the highest number of MSCRAMMs (9/15). All isolates harbored at least four or more virulence genes while SauR23 carried the highest number of virulence genes (12/19). The most prevalent toxin-encoding genes were *sem* and *seo*.

### 3.4. Congo Red Assay and Biofilm Formation Assay

All MDR-MRSA isolates were found to have the ability to form slime and biofilms with variable degrees of production (Figure 1). Among them, SauR12 and sauR91 were classified as moderate slime and biofilm-producing isolates. Although SauR110 and SauR31 were moderate slime producers, they exhibited strong biofilm production with SBF values of 1.952 and 1.284, respectively. SauR23 was identified as the strongest slime and biofilm producer, with an SBF value of 5.478.

### 3.5. Real-Time Impedance-Based Assay

**Figure 2:**
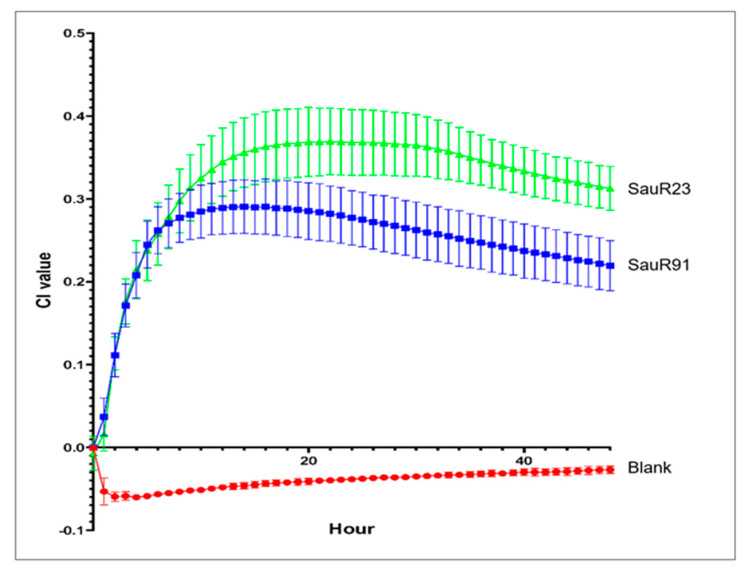
Real-time impedance monitoring of bacterial biofilm formation of SauR23 and SauR91 for 48 h. Each point in the curve corresponds with the mean (±standard deviation) calculated from three biological replicates.

Real-time impedance measurement of biofilm formation for SauR91 (moderate biofilm-producing isolate) and SauR23 (strong biofilm-producing isolate) revealed distinct phases of biofilm formation (Figure 2). After 10 h of incubation, both MRSA isolates progressed to the biofilm maturation phase. SauR23 and SauR91 reached a plateau phase in which the maximum number of bacteria adhered to the gold microelectrodes with a maximum CI value of 0.3691 and 0.2906, respectively, indicating the biofilm had matured. The maximum CI value of SauR23 was higher than that of SauR91, which confirmed that SauR23 was a strong biofilm producer, while SauR91 was a moderate biofilm producer. At 30 h, both MRSA isolates began to disperse slowly as bacterial cells were released from the matured biofilm into the medium, as indicated by the decreased rate of CI.

### 3.6. Whole Genome Sequencing

The general genomic features of SauR23 and SauR91 are summarized in Table 3. The assembled draft genome of SauR23 (accession no. JAIVEH000000000) had 124 contigs, with a total length of 2,840,058 bp, *N*_50_ value of 72,669, and an average G + C content of 32.66%. The assembled genome contained 2634 protein-coding sequences (CDS), 54 transfer RNA (tRNA) genes, and four ribosomal RNA (rRNA) genes. On the other hand, the assembled draft genome of SauR91 (NCBI accession no. JAHMGR000000000) had 51 contigs, with a total length of 2,811,984 bp, *N*_50_ value of 146,224 and an average G + C content of 32.69%. The assembled genome of SauR91 contained 2605 CDS, 58 tRNA genes, and 4 rRNA genes.

**Figure 3:**
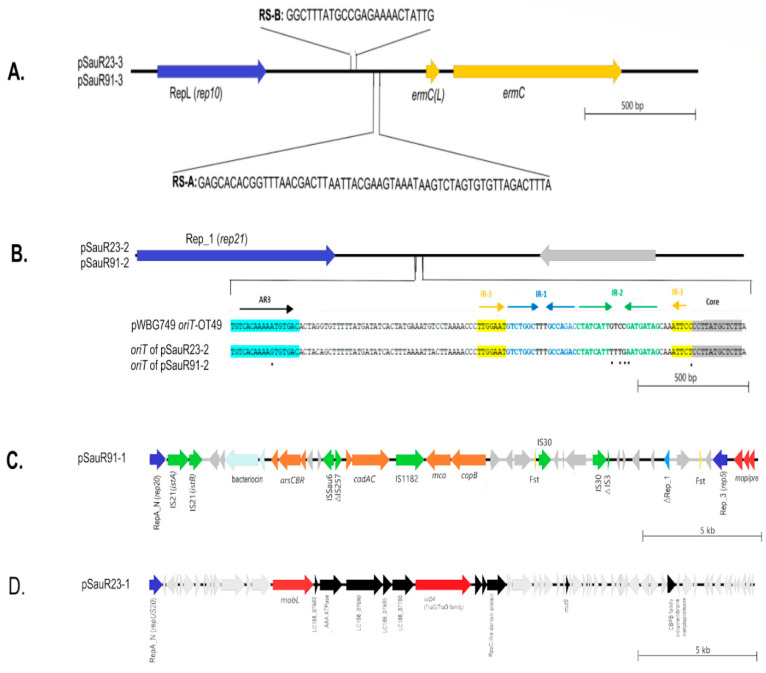
Linear genetic maps of the plasmids that were found in SauR23 and SauR91 isolates. (**A**) Linear map of pSauR23-3 and pSauR91-3 plasmids; RepL (*rep10*): replication loci, RS-A/RS-B: recombination sites, *ermc*(*L*): erythromycin resistance leader peptide and *ermC*: erythromycin resistance gene. (**B**) Linear map of pSauR23-2 and pSauR91-2 plasmids and pairwise sequence alignment of *oriT* regions of these Rep_1 plasmids (encompassing AR3, IR1, IR2, IR3, and the core sequences) with pWBG749-OT49 reference subtype, showing the nucleotide sequence similarity; AR3: accessory repeat, IR1-IR3: inverted repeats and Rep_1/*rep21*: replication region and grey arrows represent other protein-coding sequences/hypothetical proteins. (**C**) Linear map of pSauR91-1 plasmid; RepA_N (*rep20*)/Rep_3 (*rep5*): replication region, *cadAC*: cadmium resistance operon, *arsCBR*: arsenate resistance operon, *copB/mco*: copper resistance genes, *mob/pre:* mobilization gene, Fst: Toxin–antitoxin type I system, IS: insertion sequences, and grey arrows represent other protein-coding sequences/hypothetical proteins. (**D**) Linear map of pSauR23-1 conjugative plasmid; RepA_N/*repUS20:* replication region, *mobL*: mobilization gene, *virD (*TraG/TraD*):* conjugation gene, and grey arrows represent other protein-coding sequences/hypothetical proteins.

The WGS data were in concordance with the genotyping profile in which two of the isolates harbored the SCC*mec* type IV(2B) with multiple subtype target genes detected. Apart from that, they belonged to sequence type 22 (ST22) which is of the clonal complex 22 (CC 22) lineage and *spa* type t032. SauR23 hosted three plasmids designated as pSauR23-1, pSauR23-2, and pSauR23-3. Likewise, SauR91 also carried three plasmids named pSauR91-1, pSauR91-2, and pSauR91-3. The pSauR23-3 and pSauR91-3 plasmids were similar (Figure 3A) and were 2473 bp and 2600 bp in length, respectively. Both these plasmids harbor the *ermC* gene and its upstream leader peptide-coding gene, *ermC(L)*, that mediate the iMLS_B_ resistance phenotype. Their plasmid replication initiator protein belonged to the *rep10* family with a RepL conserved domain. Likewise, pSauR23-2 and pSauR91-2 are small plasmids (with sizes of 3011 bp and 3138 bp, respectively) but cryptic. They are similar in their genetic composition, compromising one replication initiator that belonged to the Rep_1 (*rep21*) family and one hypothetical protein-coding sequence (Figure 3B). A further BLASTN search revealed that these plasmids carried a short sequence (125 nts in length) similar to the pWBG749-OT49-encoded *oriT* sequence. The sequence of the Rep_1 plasmid showed a high degree of similarity (>99%) to the *S. aureus* strain JY43 plasmid pKH12 (3011 bp, accession no. EU168704.2) and other similar 3 kb cryptic staphylococcal plasmids.

pSauR91-1 is a large, potentially mobilizable plasmid (35,640 bp) with two genes encoding replicases, i.e., *rep5a* with a Rep_3 domain replicase and *rep20* with a RepA_N replicase domain, as well as a truncated Rep_1 replicase. The plasmid carried several genes that mediate resistance to heavy metals such as cadmium (*cadAC*), copper (*mco*), and arsenate (*arsAC*). pSauR91-1 is also rich with insertion sequences (ISs) with two copies of IS*30*, and one each of IS*21*, IS*1182*, and IS*Sau6* (Figure 3C). The plasmid also harbored two partial IS sequences which is indicative of a past transposition event(s): a partial IS*257* and a truncated IS*3* element (Figure 3C). pSauR91-1 showed >99% identity to *S. aureus* plasmid SAP078A (35,508 bp, accession no. GQ900430.1). pSauR91-1 also harbored a bacteriocin virulence element and two copies of the Fst type I toxin–antitoxin (TA) system. On the other hand, pSauR23-1 is a large (58,422 bp) conjugative cryptic plasmid with a single replicon belonging to the *repUS20* replicase with a RepA_N conserved domain (Figure 3D). Comparative analysis of the pSauR23-1 plasmid showed no homologous plasmids in the database, strongly suggesting that it is novel. pSauR91-1 showed only 34% similarity to its closest characterized plasmid homolog, i.e., pWBG749 (accession no. GQ900391) which is a known staphylococcal conjugative plasmid. However, the pSauR23-1 putative conjugative region differed from that of the pWBG749 conjugative transfer system. Several genes which contained conserved motifs for the MobL relaxase, the TraG/TraD-like conjugative transfer protein, and a type VI secretion system ATPase were detected within pSauR23-1.

A total of 12 and 11 AMR genes and associated genes were predicted from the WGS of SauR23 and SauR91, respectively, most of which were in concordance with their phenotypic AMR profiles (Table 4). These predicted genes were *mecA* and *blaZ* which correlated with β-lactams resistance, mutated *gyrA* (DNA gyrase/topoisomease II subunit A; S84L mutation), and *parC/grlA* (DNA topoisomerase IV subunit A; S80F mutation) which mediated ciprofloxacin resistance, *ermC* which mediated MLS_B_ resistance, the major facilitator superfamily (MFS) effux pumps encoded by *norA*, *sdrM* (both of which are known to mediate fluoroquinolone resistance), and *lmrS*, a MDR efflux pump [42]. Other possible AMR-associated regulatory genes include *mepR* (MDR efflux transporter transcriptional repressor known to repress *mepA* expression), *arlR* (response regulator ArlR), and *mgrA* (a helix-turn-helix (HTH)-type transcriptional regulator MgrA). From the CARD analysis, SauR23 has a variant of *fusA* with H457Q, L461F, and T436I mutations that is likely responsible for fusidic acid resistance. However, no specific resistance genes were detected that could confer aminoglycoside (gentamicin and amikacin) and tetracycline resistance in SauR23.

## 4. Discussion

*S. aureus* colonization typically peaks in newborn infants at 1–2 months of age and then decreases by 6 months [43]. However, hospitalized neonates have a higher rate of colonization (ranging between 3.9% and 32%) than the general neonatal population [2]. The infections caused by MRSA may cause serious consequences as it is one of the most successful pathogens with the adaptive evolutionary traits of resistance and virulence. Generally, a high-risk newborn infant population often receives broad-spectrum antimicrobial agents as initial empiric therapy based on clinical suspicion rather than on microbiological evidence of infection (positive bacterial culture results) because microbiological results are usually available only about 24 to 72 h following sampling and this is often hindered by infections caused by antimicrobial-resistant microbes including MRSA. In our present study, all five MRSA isolates were multidrug resistant and resistant to several classes of antibiotics, including β-lactams, macrolides, lincosamides, and fluoroquinolones. This suggests that selecting appropriate antimicrobial regimens for initial empiric therapy is becoming more challenging. Additionally, all five MDR-MRSA isolates in our study showed in vitro inducible clindamycin resistance by D-test. Thus far, globally, iMLSB MRSA cases have been documented with variable rates ranging from 0.65% to 76.4% [44].

In this study, both *spa* types t032 (SauR91) and t022 (SauR110) were reported and these *spa* types were closely related to the epidemic clone EMRSA-15 (MLST-ST22) which is typically resistant to fluoroquinolone [5,45]. Similar findings were also observed in this study. A retrospective case–control study in Germany reported that in 23 neonatal intensive care patients, MRSA isolates that originated from colonization or infection were of *spa* type t032 (MLST-ST22) [46]. Isolates of *spa* type t032 are common in MRSA isolates from the UK and Germany, and this clone has now been detected in Malaysia. In 2018, a study reported the detection of MRSA *spa* type t032 from the community nasal swabs of healthy adults (11.1%, 2/18) in the same eastern coast state of Malaysia as this study (i.e., Terengganu), suggesting that this clone is likely circulating in communities without exposure to healthcare settings [47]. Recently, a high prevalence of *spa* type t032 in hospitalized patients was reported from two studies conducted in central Malaysia (72.1%, 160/222) and in Terengganu (39.3%, 35/89) [27,48]. Our preliminary WGS study of 62 clinical MDR-MRSA isolates revealed that the EMRSA-15 (ST22-SCC*mec* IV [2b]) clone (67.7%) and *spa* type t032 (50%) were indeed predominant (67.7%) in our local scenario [49].

Two MDR-MRSA isolates, i.e., SauR23 and SauR31, were identified as Panton–Valentine leukocidin (PVL) positive (*lukED* and *lukPV*), which were divided into two distinct genetic clades and belonged to *spa* types t032 and t548, respectively. The t548 *spa* type was primarily associated with livestock-associated MRSA [40]. It had been found in pig farms in the United States and in traditional chicken farms in Taiwan [50,51]. These reports indicated that personnel in close contact with farmed animals increased their risk of getting livestock-associated MRSA colonization/infection [49,50], but it does not explain the finding of the t548 *spa* type in MRSA neonatal infections in our study. We postulated that breast milk may be responsible for the transmission of livestock-associated MRSA from the mother to neonates. An earlier report had inferred that breast milk was a reservoir for livestock-associated *S. aureus* (of ST398) and community-associated *S. aureus* (ST59) in China [52]. Therefore, active surveillance of MRSA carriage and transmission in both mother and neonates is needed to rule out this possible risk and enable steps to be taken to prevent such an infection.

All MDR-MRSA isolates in this study were shown to be capable of forming slime and biofilm at a moderate or strong level which helps these isolates to persist in infections. All five isolates in this study possessed the *icaAD* and *icaBC* genes but previous evidence demonstrated that deletion of the *ica* operon did not abolish biofilm-forming capacity among MRSA isolates [53]. However, the *eno*, *sasG*, and *clfB* genes which encode staphylococcal cell wall-anchored proteins were also detected in our studied isolates, supporting that additional *ica*-independent mechanisms of biofilm accumulation were present in the MRSA isolates [54]. Besides MASCRAMMs, extracellular polymeric substances including extracellular DNA and teichoic acid had also been reported to be involved in *ica*-independent mechanisms of biofilm production [54]. Subsequent real-time impedance analysis confirmed that SauR23 is a strong biofilm producer as the maximum CI value was higher than that of the moderate producer SauR91 and this result is comparable to their corresponding SBF values in the CV assay. The strong biofilm-forming isolate, SauR23, was obtained from a preterm neonate which exhibited phenotypic resistance to seven antimicrobial classes while SauR91 showed resistance to four antimicrobial classes. From the clinical data, SauR91 was isolated from the endotracheal tube of a six-month-old infant with neonatal congenital abnormalities who succumbed to the nosocomial infection after 183 days in the pediatric intensive care unit. The endotracheal intubation is a frequent procedure in intensive care units for mechanically ventilated patients and *S. aureus* in many cases show the capability to form biofilm on endotracheal tube surfaces. As the biofilm matures, it is more difficult to be treated and removed [55] and biofilm-forming MRSA resists clearance by multiple antimicrobial agents and causes persistent infections in patients [56]. Besides the production of slime and biofilms, toxin-encoding genes *sem* and *seo* were detected in all isolates and this is in agreement with a previous study that reported the *egc*-clustered enterotoxins are the most common virulence factors in MRSA [57].

In this study, WGS data revealed that both SauR23 and SauR91 carried multiple plasmids. Co-carriage of several plasmids in a single isolate is in agreement with a previous study where up to six plasmids per *S. aureus* isolate were detected [58]. The small *ermC*-carrying plasmid detected in both SauR23 (pSauR23-3) and SauR91 (pSauR91-3) is likely responsible for their iMLS_B_ phenotype. The replication initiator of this *ermC* plasmid is homologous to the replication initiator of the pSN2 family of plasmids in *S. aureus*, which are among the smallest (<3 kb) staphylococcal plasmids [59,60]. Interestingly, this pSN2 family plasmid was the only plasmid identified in SauR23 and SauR91 that harbored an antimicrobial resistance gene. Thus, for these two MDR MRSA isolates, the antimicrobial resistance genes were mainly chromosomally located. SauR91 harbored a large 35.6 kb plasmid, pSauR91-1, that encoded resistance to several heavy metals and is potentially mobilizable due to the carriage of *mob/pre* mobilization genes. On the other hand, SauR23 harbored a larger 58.4 kb putative conjugative plasmid, pSauR23-1, which is cryptic (without any identifiable resistance or virulence genes) and does not have any homologous plasmids in the database. In the absence of any selectable marker on pSauR23-1, we were unable to investigate its conjugative potential. Both SauR23 and SauR91 harbor similar small (ca. 3 kb) cryptic plasmids with the Rep_1 replicase domain. The Rep_1 replicase has relaxase potential and hence can promote plasmid dissemination besides its role in replication [61]. The pWBG749-OT49 *oriT* mimic sequence were also detected in these plasmid Rep_1 plasmids, enabling these plasmids to be mobilizable if they coexist with a self-transmissible pWBG749-type of plasmids [62].

Collectively, the drug resistance genes identified from our WGS results are consistent with various reported MDR-MRSA isolates that harbored determinants that enhanced their ability to resist antimicrobials [63,64]. These MDR-MRSA harbored *blaZ*-encoded β-lactamase to disrupt the amide bond of β-lactam ring and different families of exporters including efflux pumps such as NorA, SdrM, and LmrS to extrude drugs (e.g., macrolides, aminoglycosides, oxazolidinones, diaminopyrimidines, and phenicols) from the interior to the external environment, thus lowering their intracellular concentration [65]. Fluoroquinolone resistance in *S. aureus* is due to mutations in the target DNA topoisomerase II-encoding genes *gyrA* and *gyrB*, and the *parC*/*grlA* gene encoding DNA topoisomerase IV. Known mutations (i.e., S84L in GyrA and S80F in ParC) were detected in both sequenced MRSA isolates [63,66]. The *fusA* gene encodes the prokaryotic elongation factor G (EF-G) which is essential in protein translation and is a target for fusidic acid. Point mutations in the chromosomal *fusA* gene are known to lead to fusidic acid resistance in *S. aureus* and these were found in SauR23 [63]. However, no distinct genes were found that could account for the resistance towards gentamicin, amikacin (aminoglycosides), and tetracycline observed in SauR23. Nevertheless, efflux pumps such as the *lmrS*-encoded multidrug efflux pump, and in particular, their overexpression [63,67], may contribute to the resistance towards these two classes of antibiotics seen in SauR23, although this needs to be experimentally verified. It is interesting to note that both SauR23 and SauR91 harbored the *mepR* regulatory gene but not the corresponding *mepAB* genes which encode the MepA multidrug and toxic compound extrusion (MATE) family efflux pump. Recent work has shown that mutations in *mepR* which encode a transcriptional repressor protein, lead to the overexpression of *mepAB* genes which, in turn, leads to tigecycline resistance [68]. Overexpression of *mepA* had been shown earlier to contribute to multidrug resistance in *S. aureus* [69]. Besides *mepR*, two other regulatory genes for efflux pumps were identified in the SauR23 and SauR91 genomes, namely, *mgrA* and *arlR*. Their function or contribution to the antimicrobial resistance in these two *S. aureus* isolates are likewise, currently unknown.

Our study demonstrated the genetic and phenotypic background on five MDR-MRSA isolates and whole genome sequence analysis of two of these isolates offers better insights into the strains that infected neonates in Malaysian hospitals. Although the small sample size is an obvious limitation for this study, such knowledge of neonatal MDR MRSA has hitherto been unavailable in Malaysia. Further work on this aspect is clearly needed to improve on future diagnostic investigations of neonatal MRSA infections, particularly screening for AMR genes which should be part of the multifaceted intervention efforts in hospitals and other healthcare settings to address this issue. Genome sequencing of isolates would also help to establish if there is a circulating clone in the pediatrics ward or the neonatal intensive care units and this knowledge would indeed be beneficial for the improvement in hospital infection control programs.

## 5. Conclusions

In this study, we characterized five neonatal MDR-MRSA isolates obtained from the main tertiary hospital in Terengganu, Malaysia. All the MDR-MRSA isolates were capable of producing slime and biofilms and harbored varying profiles of MSCRAMM and virulence genes. The WGS of two isolates, SauR23 and SauR91, revealed that most antimicrobial resistance genes were chromosomally encoded except for the *ermC* gene that mediated the iMLS_B_ phenotype which was carried on a small (<3 kb) plasmid. Both isolates were typed as ST22 of the CC22 lineage and carried SCC*mec* type IV (2B). The other three isolates also harbored SCC*mec* type IV as determined by PCR. The presence of MDR-MRSA strains infecting neonates in the Terengganu hospital is of serious concern. Thus, efforts to better understand the pathogen are clearly needed with WGS giving us clues as to the lineage and carriage of AMR along with virulence genes. Expansion of the knowledge base of AMR genes via WGS is important as these metadata can be used to help improve the investigation and surveillance of MDR pathogens such as MDR-MRSA, and even more so for strains that infect neonates.

## Figures and Tables

**Table 1:** Demographic characteristics of neonates identified as infected by methicillin-resistant *Staphylococcus aureus* in Hospital Sultanah Nur Zahirah, Terengganu, Malaysia.

MRSA Isolate	SauR12	SauR23	SauR31	SauR91	SauR110
Site of isolation	Eye	Pus	Eye	Respiratory	Pus
Gender	F	M	F	F	F
Age (on MRSA collection)	Newborn	Newborn	3 weeks	6 months	Newborn
Clinical disease/MRSA infection	Other staphylococcal clinical syndromes	Nosocomial colonization	Other staphylococcal clinical syndromes	Nosocomial colonization (Endotracheal tube)	Skin and soft tissue infections (SSTI)
Underlying/comorbidities	-	Premature, mitral atresia, transposition of the great arteries (TGA), neonatal jaundice	-	Congenital abnormalities	-
Length of stay (hospitalization)	89 days	9 days	44 days	183 days	30 days
Ward	Pediatric	Neonatal intensive care unit	Neonatal intensive care unit	Pediatric intensive care unit	Pediatric
Outcome	Alive	Alive	Alive	Deceased (6 months old)	Alive
Clinical association	Hospital associated	Hospital associated	Hospital associated	Hospital associated	Hospital associated

According to the World Health Organization, a newborn infant or neonate is referring to a child under 28 days of age.

**Table 2:** Antimicrobial profiling for five methicillin-resistant *Staphylococcus aureus* isolated from neonates targeting 18 antimicrobial classes that encompass 26 antibiotics.

Antimicrobial Class	Antibiotic	Disk Concentration	Interpretative Value *			MRSA Isolate				
			Susceptible (S)	Intermediate (I)	Resistance I	Sau R12	Sau R23	Sau R31	Sau R91	Sau R110
β-lactams	Penicillin	10 U	≥29	-	≤28	**R**	**R**	**R**	**R**	**R**
	Oxacillin	1 µg	≥13	11–12	≤10	**R**	**R**	**R**	**R**	**R**
	Cefoxitin **	30 µg	≥22	-	≤21	**R**	**R**	**R**	**R**	**R**
	Cefoperazone	75 µg	≥21	16–20	≤15	**R**	**R**	**R**	**R**	**R**
Fluoroquinolones	Ciprofloxacin	5 µg	≥21	16–20	≤15	**R**	**R**	S	**R**	**R**
	Moxifloxacin	5 µg	≥24	21–23	≤20	**R**	**R**	S	**R**	**R**
Macrolides	Erythromycin	15 µg	≥23	14–22	≤13	**R**	**R**	**R**	**R**	**R**
Lincosamides	Clindamycin	2 µg	≥21	15–20	≤14	**R**	**R**	**R**	**R**	**R**
Aminoglycosides	Gentamicin	10 µg	≥15	13–14	≤12	S	**R**	S	S	S
	Amikacin	30 µg	≥18	-	<18	S	**R**	S	S	S
Folate inhibitors	Co-trimoxazole	25 µg	≥16	11–15	≤10	S	S	S	S	S
Fucidanes	Fusidic acid	10 µg	≥24	-	<24	S	**R**	S	S	S
Tetracyclines	Tetracycline	30 µg	≥19	15–18	≤14	S	**R**	S	S	S
	Minocycline	30 µg	≥19	15–18	≤14	S	S	S	S	S
	Doxycycline	30 µg	≥16	13–15	≤12	S	S	S	S	S
Glycylcyclines	Tigecycline	15 µg	≥18	-	<18	S	S	S	S	S
Phenicols	Chloramphenicol	30 µg	≥18	13–17	≤12	S	S	S	S	S
Monoxycarbolic acid	Mupirocin	5 µg	≥14	-	≤13	S	S	S	S	S
Ansamycins	Rifampin	5 µg	≥20	17–19	≤16	S	S	S	S	S
Aminocoumarin	Novobiocin	5 µg	≥16	-	≤12	S	S	S	S	S
Glycopeptides	Vancomycin	30 µg	≥17	15–16	≤14	S	S	S	S	S
	Teicoplanin	30 µg	≥14	11–13	≤10	S	S	S	S	S
Oxazolidinones	Linezolid	30 µg	≥21	-	<21	S	S	S	S	S
Phosphonic acids	Fosfomycin	200 µg	≥16	13–15	≤12	S	S	S	S	S
Streptogramins	Quinupristin-dalfopristin	15 µg	≥19	16–18	≤15	S	S	S	S	S
Anti-MRSA cephalosporins	Ceftaroline	30 µg	≥24	21–23	≤20	S	S	S	S	S
No. AMR Classes ^‡^						4	3	7	4	4
D-test ^‡‡^						+	+	+	+	+

* S, susceptible; I, intermediate; R, resistance; interpretive breakpoints according to CLSI [24] and EUCAST [25]. ** Cefoxitin resistance = MRSA determinant. ^‡^ No. AMR classes, numbers of resistance to antimicrobial classes; ≥3 AMR classes = multidrug resistance (MDR) [26]. ^‡‡^ D-test positive (+) = iMLSB phenotype.

**Table 3:** Draft genome of SauR23 and SauR91.

Feature	SauR23	SauR91
Circular genome map * 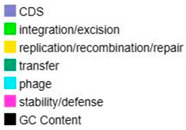	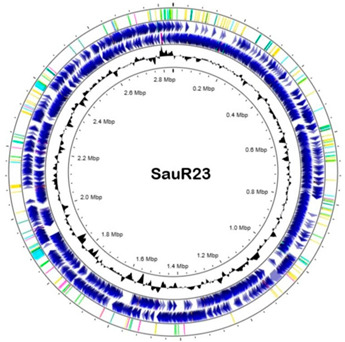	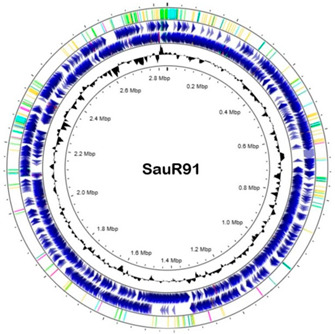
Genome NCBI accession no.	JAIVEH000000000	JAHMGR000000000
Total length (bp)	2,840,058	2,811,984
No. of contigs	124	51
GC (%)	32.66	32.96
N_50_	72,669	146,224
Protein coding sequences	2634	2605
No. of tRNA genes	54	58
No. of rRNA genes	4	4
Plasmid (NCBI accession no.)/size (bp)	pSauR23-1 (JAIVEH010000014.1)/58,422	pSauR91-1 (JAHMGR010000023.1)/35,640
	pSauR23-2 (JAIVEH010000068.1)/3011	pSauR91-2 (JAHMGR010000027.1)/3138
	pSauR23-3 (JAIVEH010000073.1)/2473	pSauR91-3 (JAHMGR010000029.1)/2600

* Circular genome maps of *Staphylococcus aureus*, from the outer to the inner circle: (1) genomic features detected in the respective genomes: integration/excision sequences were showed in light green color, replication/recombination/repair genes represented in yellow, transfer elements are showed in dark green, phage showed in light blue, stability/defense in pink; (2) forward strand CDSs; (3) forward strand CDSs (4) GC content.

**Table 4:** Resistance genes identified from the genomes of MRSA isolates SauR23 and SauR91 with their corresponding resistance phenotypes.

Resistance Phenotype	Antimicrobial Class	Resistance Genotype	Mechanism
Penicillin	β-lactams	*blaZ* family	Antibiotic inactivation enzyme
Cefoxitin, oxacillin, cefoperazone	β-lactams	*mecA*	Antibiotic target alteration
Ciprofloxacin, moxifloxacin	Fluoroquinolones	*gyrA* (mutation, S84L)	Amino acid change in GyrA (DNA gyrase/Topoisomerase II)
		*parC/grlA* (mutation, S80F)	Amino acid change in ParC (Topoisomerase IV)
		*norA*	NorA MFS efflux pump
		*sdrM*	SdrM MFS efflux pump
Erythromycin	Macrolide	ermC *	Antibiotic target alteration
		*lmrS*	Multidrug resistant MFS efflux pump
Clindamycin	Lincosamide	ermC *	Antibiotic target alteration
		*lmrS*	Multidrug resistant MFS efflux pump
Fucidic acid (SauR23)	Fucidanes	*fusA* (mutations)	Antibiotics target alteration
Gentamicin, amikacin (SauR23)	Aminoglycosides	*lmrS*	Multidrug resistant MFS efflux pump

* Located in plasmids pSauR23-3 (JAIVEH010000073.1) and pSauR91-3 (JAHMGR010000029.1) and responsible for their iMLS_B_ phenotype; S84L = serine to leucine substitution at amino acid 84; S80F = serine to phenylalanine substitution at amino acid 80; MFS, major facilitator superfamily.

## Data Availability

The draft of genomes and plasmids of *Staphylococcus aureus* SauR23 and SauR91 have been deposited in the GenBank database. The draft genome of SauR23 is under the accession no. JAIVEH000000000 along with plasmids of pSauR23-1 (JAIVEH010000014.1), pSauR23-2 (JAIVEH010000068.1), and pSAuR23-3 (JAIVEH010000073.1), whereas the genome of SauR91 is under the accession no. JAHMGR000000000 along with plasmids of pSauR91-1 (JAHMGR010000023.1), pSauR91-2 (JAHMGR010000027.1), and pSAuR91-3 (JAHMGR010000029.1).
